# Wahlen in Pandemiezeiten – ein juristisches Risikogebiet

**DOI:** 10.1007/s41358-021-00259-2

**Published:** 2021-04-27

**Authors:** Heike Merten

**Affiliations:** Institut für Deutsches und Internationales Parteienrecht und Parteienforschung (PRuF), Heinrich-Heine-Universität Düsseldorf, Gebäude 37.03, Universitätsstraße 1, 40225 Düsseldorf, Deutschland

## Einführung

Die regelmäßige und ordnungsgemäße Durchführung von Wahlen ist die Grundvoraussetzung für eine funktionierende Demokratie. Wahlen müssen daher auch in Pandemiezeiten durchgeführt werden. In der Corona-Krise waren 2020 zunächst die Stichwahl zur bayerischen Kommunalwahl und im bevölkerungsstärksten Bundesland Nordrhein-Westfalen die Kommunalwahlen betroffen. Im Super-Wahljahr 2021 stehen die Kommunalwahlen in Hessen und Niedersachsen, Landtagswahlen in Baden-Württemberg, Rheinland-Pfalz, Sachsen-Anhalt, Mecklenburg-Vorpommern, Thüringen und Berlin und darüber hinaus auch die Bundestagswahl an. Der Beitrag gibt einen Überblick, wie Wahlen auch in Pandemiezeiten rechtssicher durchgeführt werden können. Von der Kandidatenaufstellung über den Wahlkampf bis zum eigentlichen Wahlakt gilt es dabei, einige juristische Hürden zu nehmen.

## Wahlverschiebung

Eine Verschiebung von Wahlen auf einen Zeitpunkt nach der Pandemie und damit eine Verlängerung der laufenden Wahlperioden ist keine realistische Option. Zum einen ist nicht vorhersehbar, wann die Pandemie vorbei sein wird. Zum anderen sehen die Verfassungen zum Schutz der Demokratie derartige Wahlverschiebungen aus einer laufenden Legislaturperiode heraus nicht vor.

Die Periodizität von Wahlen gehört zu den notwendigen Bestandteilen einer Demokratie, die immer nur eine „Herrschaft auf Zeit“ (BVerfGE 119, 247, Rn. 47) sein kann. Dieses änderungsfeste (Art. 79 Abs. 3 GG) Kernelement des Demokratieprinzips gilt über Art. 28 Abs. 1 GG auch in den Ländern. Es sichert, dass die Legitimation von Amtsträgern in regelmäßigen Abständen durch das dazu berufene Volk erneuert wird. Die Dauer der regulären Legislaturperiode der Parlamente ist daher verfassungsrechtlich festgeschrieben und eine Veränderung lediglich im Wege einer Verfassungsänderung möglich. Eine Verkürzung oder Verlängerung der laufenden Wahlperiode durch das amtierende Parlament im Wege einer Verfassungsänderung wird von überwiegender Auffassung in der Rechtswissenschaft, im Gegensatz zu einer Veränderung der Dauer künftiger Wahlperioden, allerdings ausgeschlossen (Hahlen 2017, § 16 Rn. 10 m. w. N.). Den Abgeordneten eben dieser laufenden Wahlperiode fehle es an der notwendigen demokratischen Legitimation, ihre eigene Amtszeit zu verlängern.

Diese für die Bundes- und Landesebene herausgearbeiteten verfassungsrechtlichen Kriterien finden auf der kommunalen Ebene lediglich in ihren Grundsätzen (Art. 28 Abs. 1 S. 1 GG) Anwendung. Auf der kommunalen Ebene ist die Wahlperiode nicht verfassungsrechtlich, sondern ausschließlich einfachgesetzlich festgeschrieben. Zur Anpassung der Laufzeit der Wahlperiode ist damit ausschließlich der Landtag als Gesetzgeber berufen, der so zwar nicht über die Verlängerung seiner „eigenen“ Legislaturperiode entscheidet, aber dennoch an die demokratischen Vorgaben gebunden ist. Die Landesverfassungsgerichte halten dies für verfassungsgemäß, „wenn die Verlängerung im Verhältnis zur Dauer der regulären Wahlzeit gering ist und durch wichtige Gründe des Gemeinwohles gerechtfertigt wird“ (VerfGH NRW, Beschluss v. 30.06.2020 Az. 63/20.VB-2- Rn. 74  m. w. N.). Die vom Verfassungsgerichtshof NRW in den Blick genommene wertende Abwägung des Landesgesetzgebers zugunsten der termingerechten Durchführung der Kommunalwahlen im September 2020 war aber auch nach seiner Auffassung die verfassungsrechtlich „richtige“ Entscheidung (VerfGH NRW, Beschluss v. 30.06.2020 VerfGH- 63/20.VB-2- Rn. 75). Eine Verlängerung der laufenden Wahlperiode wäre vor diesem Hintergrund mit deutlicher Rechtsunsicherheit behaftet.

Ist mithin eine Verschiebung der Bundestags- und Landtagswahlen nicht und auf kommunaler Ebene kaum möglich, muss die Vorbereitung und Durchführung der Wahlen näher in den Blick genommen werden.

## Kandidatenaufstellung

Der erste wichtige Schritt zur Wahlvorbereitung ist der Akt der Aufstellung der Kandidaten, denn ohne Kandidaten keine Wahl. Die Kandidatennominierung ist eine unentbehrliche Voraussetzung für die Durchführung der Wahl und muss demokratischen Grundsätzen entsprechen. Die Nominierung erfolgt nach den jeweiligen Wahlgesetzen in Mitglieder- oder Delegiertenversammlungen unter Anwesenden (Merten 2019, S. 119 ff.). Da solche Präsenzveranstaltungen in Zeiten einer Pandemie eine nicht unerhebliche Gesundheitsgefahr mit sich bringen könnten, suchte man nach Lösungen, wie eine pandemiekonforme Kandidatenkür möglich gemacht werden kann.

Der Deutsche Bundestag hat mit § 52 Abs. 4 BWahlG die Lösung darin gesehen, das Bundesinnenministerium zu ermächtigen, im Falle einer Naturkatastrophe oder ähnlicher Ereignisse durch Rechtsverordnung von den gesetzlichen Bestimmungen zur Kandidatenaufstellung abzuweichen. Der Erlass der Rechtsverordnung bedarf der Zustimmung des Bundestages, der zuvor feststellen muss, dass die Durchführung von Präsenzveranstaltungen ganz oder teilweise unmöglich ist. Diese notwendige Feststellung hat der Bundestag am 14. Januar 2021 getroffen und das Bundesinnenministerium in der Folge die sog. COVID-19-Wahlbewerberaufstellungsverordnung^1^ erlassen.

Eine Absicherung der ordnungsgemäßen Durchführung der Bundestagswahl ist richtig und notwendig, allerdings ist der gewählte Weg durchaus zweifelhaft. Dies in mehrfacher Hinsicht. Zum einen darf die konkrete Ausgestaltung wesentlicher wahlrechtlicher Belange nicht der Exekutive überlassen werden. Das Parlament muss diese grundlegenden Fragen der Aufstellung von Wahlbewerber nach dem Grundsatz des Parlamentsvorbehaltes selbst regeln (Michl 2021; von Notz 2020). Nach der ermächtigungsauslösenden Feststellung der Unmöglichkeit von Versammlungen durch den Bundestag muss dieser der Rechtsverordnung der Exekutive dann noch zustimmen. Warum der Gesetzgeber dieses dreistufige Verfahren wählt und nicht die Gesetzesänderung gleich selbst vornimmt, ist unverständlich. Zum anderen ist fraglich, wie der Bundestag die Unmöglichkeit der Durchführung von Versammlungen feststellen kann, wenn diese Unmöglichkeit rein tatsächlich überhaupt nicht eintreten kann. Eine Versammlung ist unter Pandemiebedingungen natürlich möglich, allerdings mit einem gewissen Gesundheitsrisiko verbunden. Es liegt somit kein Fall der Unmöglichkeit vor, sondern lediglich eine gesundheits- und verfassungspolitische Abwägungsentscheidung. Rein rechtlich können Wahlversammlungen, aus guten Gründen, nicht so einfach verboten werden. Daher werden in den Coronaschutzverordnungen der Bundesländer die notwendigen Parteiversammlungen immer ausgenommen. Die festgestellte Unmöglichkeit kann es somit nicht geben. Daneben ist auch die inhaltliche Bestimmtheit der Verordnungsermächtigung durchaus diskutierbar (S. Schönberger 2021).

Aber wie hat nun das Bundesinnenministerium konkret seinen relativ weiten Gestaltungsspielraum genutzt? Die Verordnung ermöglicht, von der Pflicht zur Durchführung von Präsenzversammlungen zur Wahl der Bewerber für Kreiswahlvorschläge und Landeslisten und der Vertreter für Vertreterversammlungen zur Bundestagswahl 2021 abzuweichen und Versammlungen mittels elektronischer Kommunikation durchzuführen. Parteien können außerdem in ihrer Satzung festgelegte Mindestteilnehmerzahlen ohne Satzungsänderung verringern, um die Beschlussfähigkeit sicherzustellen. Die Schlussabstimmung über die Kandidaten, die Listen und die Vertretenden für die Vertreterversammlungen muss weiterhin per Urnen- oder Briefwahl oder als Kombination aus Urnen- und Briefwahl durchgeführt werden. Die Wahlvorschlagsberechtigten entscheiden frei, ob und wie sie von den Möglichkeiten der COVID-19-Wahlbewerberaufstellungsverordnung Gebrauch machen. Sie können auch weiterhin Präsenzversammlungen durchführen.

Die Verlagerung des Versammlungsgeschehens und damit eben auch der Vorauswahl der Kandidaten und Listenplatzierung in den digitalen Bereich ist unter demokratischen Gesichtspunkten nicht ganz unproblematisch (C. Schönberger 2016). Zulässig ist dies allenfalls in sehr engen Grenzen, etwa aus zwingenden Gründen des Gesundheitsschutzes. Noch deutlich restriktiver sind die verfassungsrechtlichen Vorgaben in Hinblick auf die Willensbildung mit demokratischen Mitteln. Auch für die Vorauswahl der Kandidaten als wesentliche Wahlvorbereitungshandlung findet der vom Bundesverfassungsgericht aufgestellte Maßstab für die staatlichen Wahlen und damit das Demokratieprinzip sowie die Wahlrechtsgrundsätze des Art. 38 GG Anwendung (BVerfGE 89, 243 (251 f.)). Danach müssen alle wesentlichen Schritte der Wahl auch einer öffentlichen Überprüfbarkeit unterliegen (BVerfGE 123, 39 ff.). An einer solchen Überprüfbarkeit der Ergebnisermittlung, die bei der Kandidatenaufstellung in Parteien durch die Parteimitglieder erfolgen können muss, fehlt es aber bei einer rein elektronischen Wahl, da bei elektronischen Abstimmungssystemen die konkrete Abstimmungssituation nicht nachvollzogen werden kann. Aus diesem Grund sind Kandidatenaufstellungen, die sich im Wesentlichen über derartige elektronische Abstimmungssysteme vollziehen, verfassungsrechtlich höchst problematisch. Die Verordnung sieht daher auch vor, dass jedenfalls die endgültige Abstimmung über einen Wahlvorschlag und damit „die verbindliche Abstimmung über denjenigen Kandidaten, den die Mehrheit im elektronischen Abstimmungsverfahren als Wahlkreisbewerber gewählt hat, oder über die im elektronischen Abstimmungsverfahren durch die Mehrheit aufgestellte Landesliste mit sämtlichen Bewerbern und deren Reihenfolge“ (Bundeswahlleiter 2021, S. 24) nur durch eine schriftliche mit Stimmzetteln geheim durchzuführende Abstimmung der Stimmberechtigten zulässig ist. Verkannt wird dabei allerdings, dass schon die „unverbindliche Vorauswahl“ auf elektronischem Wege so wesentlichen Einfluss auf die verbindliche Schlussabstimmung hat, dass auch dort vom Grundsatz der Öffentlichkeit der Wahl nur schwer Abstand genommen werden kann. Besonders deutlich wird dies bei der Listenaufstellung, wo durch die „unverbindliche Vorauswahl“ die freie Auswahlmöglichkeit der wahlberechtigten Parteimitglieder unter den Bewerbern und damit der dem demokratischen Prinzip auch innewohnende Minderheitenschutz beschnitten wird. Eine Blockwahl, die den Wahlberechtigten keinerlei Einfluss auf die konkrete Zusammensetzung und Reihenfolge der Listenkandidaten mehr belässt, ist keine Wahl im eigentlichen Sinne, sondern eine reine Bestätigung.

Daneben ist auch die Gleichheit der Teilnahme an der innerparteilichen Kandidatenaufstellung, die im Demokratieprinzip und der Wahlgleichheit wurzelt, durch eine praktisch verbindliche (Voraus‑)Wahl der Kandidaten auf elektronischem Wege in besonderer Weise gefordert. Es ist zwingend sicherzustellen, dass alle Parteimitglieder in gleicher Weise, unabhängig vom Vorhandensein privater elektronischer Endgeräte oder einem leistungsfähigen privaten Internetanschluss, an einer elektronischen Kandidatenauswahl teilnehmen könnten.

Einige Bundesländer folgen diesem problematischen Lösungsweg des Bundesgesetzgebers und planen ebenfalls, eine Verordnungsermächtigung in das Landeswahlgesetz aufzunehmen, so etwa NRW (LT-Drs. 17/11681), dies obwohl für die Durchführung der Kommunalwahlen 2020 richtigerweise pandemiebedingte Anpassungen noch durch ein Gesetz^2^ vorgenommen wurden. Länder wie beispielweise Thüringen gehen erfreulicherweise den Weg, die wesentlichen Fragen in einem Gesetz regeln zu wollen. Bei der derzeit laufenden Nominierung der Wahlkreiskandidaten ist der Presse zu entnehmen, dass die Parteien nahezu ausschließlich auf pandemiekonforme Präsenzveranstaltungen setzen, wohl auch, um eine rechtssichere Kandidatennominierung zu gewährleisten.

## Wahlzulassung und Unterstützungsunterschriften

Nach einer erfolgreichen Aufstellung der Kandidaten gilt es, die offizielle Zulassung zur Wahl zu erlangen. Dafür müssen Parteien und Wählergruppen, die bisher nicht ununterbrochen vertreten waren, sog. Unterstützungsunterschriften beibringen (z. B. §§ 20 Abs. 2 S. 2; 27 Abs. 1 S. 2 BWahlG). Die Unterzeichnungswilligen müssen in den Unterstützungsformblättern ihre Daten (Name, Geburtsdatum, Anschrift) persönlich und handschriftlich eintragen (z. B. § 34 Abs. 4; § 39 Abs. 3 BWahlO). Die Anzahl der beizubringenden Unterstützungsunterschriften von Wahlberechtigten ist gestaffelt. Mit diesem Erfordernis von Unterstützungsunterschriften wird zwar auch schon unter regulären Bedingungen die Chancen- und Wahlrechtsgleichheit beeinträchtigt. Die wahlrechtliche Zulassungsbeschränkung ist allerdings verfassungsrechtlich gerechtfertigt, da sie auf einem zwingenden Grund beruht. Regelungen, die die Zulassung eines Wahlvorschlags von der Beibringung einer Mindestzahl von Unterstützungsunterschriften abhängig machen, dienen der Sicherung der Ernsthaftigkeit des Wahlvorschlags, mit dem Ziel, letztlich auch einer Stimmenzersplitterung entgegenzuwirken. Die Legitimität dieses Anliegens ist in der verfassungsgerichtlichen Rechtsprechung grundsätzlich anerkannt (BVerfGE 81 (96 f.) 82, 353 (364); 114, 107, (115 ff.)).

Diese grundsätzliche verfassungsrechtliche Zulässigkeit, eine Wahlteilnahme von der Beibringung von Unterstützungsunterschriften abhängig zu machen, steht allerdings unter dem „Rechtfertigungs-“Vorbehalt einer rein tatsächlich auch möglichen Umsetzbarkeit, dies sowohl in zeitlicher wie auch in organisatorischer Hinsicht. Dabei muss im Blick behalten werden, dass die gesetzliche Regelung bereits jetzt von einer recht hohen Anzahl beizubringender Unterstützungsunterschriften ausgeht und den kleinen Parteien einen erheblichen organisatorischen Aufwand abverlangt (Merten 2020b).

Gilt dies schon unter „normalen Bedingungen“, so empfiehlt es sich, in Pandemiezeiten im Interesse der Wahrung der politischen Chancengleichheit hinsichtlich der Unterstützungsunterschriften deutliche Milderungen einzuführen oder von einer Unterschriftenklausel gänzlich Abstand zu nehmen. So regte etwa das Verfassungsgericht Berlin eine Absenkung auf 20 bis 30 % des Niveaus vor der Corona-Krise an (VerfGH Berlin, Beschlüsse v. 17.03.2021, Az. VerfGH 4/21, VerfGH 20 und 20 A/21; VerfGH NRW, Urteil v. 30.06.2020, Az. 63/20.VB‑2; VerfGH Rheinland-Pfalz, Beschlüsse v. 28.01.2021, Az. VGH O 82/20 und VGH A 83/20).

## Wahlkampf

Die aktuelle Corona-Pandemie und die damit einhergehenden Kontaktbeschränkungen führen faktisch zu massiven Einschränkungen der Wahlkampfaktivitäten im Straßen- und Haustürwahlkampf. Infostände und Flugblattverteilung in Fußgängerzonen wie auch der persönliche Wahlkampfbesuch sind bei Überschreitung bestimmter Inzidenzwerte praktisch sogar unmöglich. Dies gilt auch für Saal- oder Bühnenveranstaltungen.

Eine kritische Prüfung dieses faktischen Wahlkampfverbotes im öffentlichen Raum muss an den einschlägigen Verfassungsbestimmungen (Art. 21 und 38 GG) für den Schutz von Wahlwerbung ansetzen (Merten 2020a, S. 12). Daneben werden auch Freiheits- (Art. 5 GG) und Gleichheitsaspekte (Art. 3 GG) mit einbezogen (BVerwGE 47, 280 (284)).

Mit dem Wahlvorschlagsrecht untrennbar verbunden ist die Möglichkeit, für die eigenen Kandidaten werben zu dürfen. Das Recht auf Wahlkampfführung muss unter Wahrung der Chancengleichheit allen Trägerinnen und Trägern der zugelassenen Wahlvorschläge gleichermaßen zukommen (Merten 2020a, S. 12).

Dieses verfassungsrechtliche Recht auf chancengleiche Wahlkampfführung kann nur zum Schutz kollidierender Verfassungsgüter eingeschränkt werden. Ob und in welcher Weise Einschränkungen tatsächlich zu rechtfertigen sind, ist dann eine Frage der Abwägung im Einzelfall, die insbesondere auch dem Chancengleichheitsgrundsatz Rechnung tragen muss.

Grundsätzlich bleibt der kontaktlose Wahlkampf auch in Corona-Zeiten möglich (VerfGH NRW, Beschluss vom 30.06.2020, VerfGH – 76/20 Rn. 57), etwa durch Plakatierungen, Postwurfsendungen oder auch den selbst organisierten Einwurf von Flyern in Briefkästen und durch Wahlwerbesendungen im öffentlich-rechtlichen Rundfunk. In Rechnung zu stellen ist aber auch, dass sich der Wahlkampf in den letzten Jahren zunehmend in den digitalen Raum verlagert hat. Sofern Wahlkampfaktivitäten Corona-bedingt eingeschränkt sind, verbleiben jedenfalls noch zahlreiche Möglichkeiten der kontaktlosen Werbung, so dass grundsätzlich ein Wahlkampf möglich bleibt und möglich bleiben muss. Zwar geht damit ein Vorteil für etablierte Parteien gegenüber erstmals Antretenden einher und auch im Zweifel ein Vorteil für Amtsinhaber. Dies ist aber kein spezifisch Corona-bedingtes Problem. Dieses Ungleichgewicht der Erfolgschancen der Wahlwerbung begleitet generell jeden Wahlkampf (Merten 2020a, S. 13). Durch Pandemie-Maßnahmen bestehende Wahlkampfbeschränkungen treffen jedenfalls alle Parteien gleichermaßen und gewährleisten – nur unter erschwerten Bedingungen – einen chancengleichen Wahlkampf.

## Wahlakt

Beim eigentlichen Wahlakt müssen die geschriebenen Wahlrechtsgrundsätze aus Art. 38 Abs. 1 S. 1 GG sowie der vom Bundesverfassungsgericht entwickelte ungeschriebene Wahlrechtsgrundsatz der Öffentlichkeit der Wahl eingehalten werden. Danach müssen alle wesentlichen Schritte der Wahl öffentlicher Überprüfbarkeit unterliegen (BVerfGE 123, 39 ff.). Gewährleistet werden kann die Einhaltung aller fünf Wahlrechtsgrundsätze bei der als Regelfall gedachten Urnenwahl unter Anwesenden im Wahllokal am Wahltag. Das Bundesverfassungsgericht spricht daher auch von der Urnenwahl als dem „verfassungsrechtlichen Leitbild“ (BVerfGE 134, 25 Rn. 16). Eine traditionelle Urnenwahl im Wahllokal birgt jedoch in Pandemiezeiten durchaus Risiken und so wurde über die Option einer generellen Briefwahl nachgedacht (Orlowski und Pohlmann 2020; Orlowski 2020).

Die Briefwahl hingegen ist ein „privatisierter Wahlakt“ (Hahlen 2017, § 36 Rn. 4) bei dem die öffentliche Kontrolle der Stimmabgabe, aber auch die Wahlfreiheit und das Wahlgeheimnis zurückgenommen ist. Das Bundesverfassungsgericht erachtet sie dennoch ausnahmsweise für zulässig, um „eine möglichst umfassende Wahlbeteiligung zu erreichen und damit dem Grundsatz der Allgemeinheit der Wahl Rechnung zu tragen.“ (BVerfGE 134, 25 (Rn. 13)). Für die Zulässigkeit einer reinen Briefwahl müssen daher neben der Allgemeinheit der Wahl noch andere Verfassungsgüter von erheblichem Gewicht für eine verfassungskonforme Einschränkung der Wahlrechtsgrundsätze streiten. Angeführt wird dafür die aus Art. 2 Abs. 2 S. 2 GG folgende Schutzpflicht des Staates für die Gesundheit der Bürgerinnen und Bürger. Infektionen mit dem Coronavirus bei einem öffentlichen Wahlvorgang auszuschließen, sei ein verfassungsrechtlich legitimer Grund für die Durchführung einer Wahl als generelle Briefwahl (Lindner 2020). Der Aspekt des Gesundheitsschutzes tritt so zum verfassungsrechtlichen Ziel einer möglichst hohen Wahlbeteiligung hinzu.

Fraglich ist vor diesem Hintergrund, ob der Gesundheitsschutz am Wahltag tatsächlich ausschließlich durch eine reine Briefwahl gewährleistet werden kann (Orlowski 2020).

Solange es möglich ist, eine Wahl an der Urne unter Einhaltung des Infektionsschutzes sicherzustellen, ist dies zur Sicherung der Wahlrechtsgrundsätze verfassungsrechtlich geboten und eine reine Briefwahl verfassungsrechtlich kaum zu rechtfertigen. Denn auch bei einem grundsätzlichen Festhalten an der Urnenwahl steht gerade für Risikopersonen die Möglichkeit der Briefwahl oder auch der vorgezogenen Urnenwahl als Alternative zur Verfügung.

Im Rahmen der wahlordnungsrechtlichen Vorschriften ist für ein hohes Maß an Infektionsschutz zu sorgen: z. B. sollten im Wahllokal für jeden Wahlberechtigten eigene Schreibgeräte bereitstehen; Wahlkabinen und -urnen regelmäßig desinfiziert werden und auch die sonstigen Hygiene- und infektionsschutzrechtlichen Maßnahmen sind selbstverständlich einzuhalten. Die Maskenpflicht gilt natürlich auch im Wahllokal. Das in den Wahlgesetzen normierte Verhüllungsverbot für Wahlorgane (z. B. § 10 Abs. 2 S. 2 BWahlG) muss für Pandemiezeiten eine Ausnahmeregelung erfahren.

## Ausblick

In Pandemiezeiten rechtssicher durch das Superwahljahr 2021 zu kommen, ist sicherlich eine besondere Herausforderung. Ist die Wahlvorbereitung und der Wahlkampf erst einmal geschafft, steht auch einer Urnenwahl wohl nichts mehr im Wege. Denn solange in Impfzentren geimpft werden kann, solange kann jedenfalls auch in Wahllokalen gewählt werden.

## Footnotes

1 Verordnung des Bundesministeriums des Innern, für Bau und Heimat, Verordnung über die Aufstellung von Wahlbewerbern und die Wahl der Vertreter für die Vertreterversammlungen für die Wahl zum 20. Deutschen Bundestag unter den Bedingungen der COVID-19-Pandemie (COVID-19-Wahlbewerberaufstellungsverordnung), vom 28. Januar 2021 (BGBl. I S. 115).

2 Gesetz zur Durchführung der Kommunalwahlen 2020, GV. NRW. Vom 02.06.2020, S. 357 ff.
